# Toll-Like Receptor Mediated Regulation of Breast Cancer: A Case of Mixed Blessings

**DOI:** 10.3389/fimmu.2014.00224

**Published:** 2014-05-16

**Authors:** Nabiha Yusuf

**Affiliations:** ^1^Department of Dermatology, University of Alabama at Birmingham, Birmingham, AL, USA

**Keywords:** toll-like receptor, breast cancer, chemotherapy, vaccine, innate immunity

## Introduction

Breast cancer is the most common type of cancer in American women after skin cancer. It is also the second deadliest cause of fatalities in women, exceeded only by lung cancer (1). There is a lot of variation within the tumors of an individual and also between the tumors of different individuals. These differences account for assessing therapeutic resistance and progression of disease. It is a challenge to characterize these tumors and design effective therapies to control their progression (2). There is a dynamic interaction between the tumors and the immune system, which determines the fate of their existence (3). Most tumors arise as a result of genetic and epigenetic changes that occur within an individual. These changes are often followed by inflammation that helps in the recruitment of inflammatory cells, fibroblasts, and endothelial cells in the microenvironment of host tumor cells. The interaction of tumor cells and the cells of the tumor microenvironment determines the invasive potential of the tumors (4).

## Innate Immune Responses and Cancer

The cells of the innate immune system, namely macrophages, dendritic cells (DCs), natural killer (NK), NKT, and γδT-cells, play a critical role in hot immune responses against host tumors by various mechanisms (5). Adaptive immune responses play a critical role in elimination of tumor cells by generating more specific tumor immunity and immunological memory (5). Thus, there is a constant interaction between the innate and adaptive arms of the host immune system to generate a strong immune response to detect and eliminate the pathogens and mutated cells before they become tumors (6). Pathogen-associated molecular patterns (PAMPs) are recognition molecules that are associated with groups of pathogens. Damage-associated molecular patterns (DAMPs) are endogenous molecules created upon tissue injury. Both these patterns signal the threat of either infection or injury to the organism and are recognized by a family of innate immune system called the Pattern recognition receptors (PRRs) (7–10). Toll-like receptors (TLRs) are the most well studied among the members of the cellular receptors and are known to play an important role in bridging innate and adaptive immune responses in cancer (6). The innate immune responses generated by TLRs are known to suppress the function of regulatory T-cells (T-regs) by breaking tolerance and enhancing immune responses against cancers (5, 11–13). Signaling through TLR activates DCs and macrophages to secrete IL-12, a cytokine that directs the adaptive immune response toward a Th1 phenotype (14). TLRs are not only expressed on immune cells but they are also expressed on cancer cells. The expression of TLRs on the cell type can have different consequences (15). Studies suggest that TLR signaling in tumor cells promote tumor angiogenesis and metastasis. Activation or TLRs by DAMPs released by tissue damage can attract more inflammatory cells to cause chronic inflammation leading to tumor progression (16–18).

## Toll-Like Receptors and Immune Surveillance in Breast Cancer

Several TLR agonists have been demonstrated to produce anti-tumor effects in breast cancer (11). Some analogs of nucleic acids that activate TLR7 and TLR9 have been used in clinical trials to improve anti-tumor immune response against solid tumors. Additionally, TLR signaling has been shown to decrease or de-repress the effects of regulatory T-cells on DCs or CD8+ T-cells (19, 20). Among the tumor-infiltrating lymphocytes, a major population (70–90%) of gammadelta (γδ) cells called γδ1 T-cells was shown to inhibit naive and effector T-cell responses and block DC maturation and function (21). γδ1 regulatory T-cells reside naturally in the epithelial tissues and can easily migrate to normal or malignant epithelium. These cells can possibly expand by direct presentation of antigens by tumor cells (21). The immunosuppressive activity of these cells could be reversed by human TLR8 ligands both *in vitro* and *in vivo*. These cells required MyD88, TRAF6, IKKalpha IKKbeta, and p38alpha molecules in gammadelta1 cells to respond to TLR8 ligands (21). In a model of human HER-2/neu(+) breast cancer (neu-transgenic mice), topical treatment with a TLR7 agonist, imiquimod, showed significant regression of spontaneous breast cancers. Analysis of gene expression data from the tumors of these mice revealed that treatment with imiquimod resulted in high levels in addition to TNF-alpha and IFN-gamma. The anti-tumor effect of imiquimod was significantly enhanced by blocking IL-10, thereby increasing survival in these mice. Thus, IL-10 induction maybe a self-regulatory mechanism used by the TLR agonists to control excessive inflammation (22).

Other TLRs expressed on the immune cells have also been reported to improve the efficacy of tumor vaccines or enhancement of chemotherapy of breast tumors by enhancing anti-tumor immune responses. When polysaccharide krestin (PSK), a TLR2 agonist was orally consumed in neu-transgenic mice, it significantly inhibited breast cancer growth by its action on the CD8 (+) T-cell and NK cells but not CD4 (+) T-cells (23). In another study, another TLR2 agonist polysaccharide A (PSA) was shown to cause inhibition of immune responses by production of IL-10 and regulatory T-cells (24). Thus, TLR2 stimulation on immune cells may also have opposing immune effects as in the case of PSA and PSK. Nitrogen bisphosphonates (NBPs) have been shown to cause a rapid influx of neutrophils and monocytes that was dependent via myeloid differentiation primary response gene 88 (MyD88), a downstream adaptor molecule involved in TLR and IL-1 signaling. Using bone marrow chimeras, it was demonstrated that this acute inflammatory response was partially dependent on TLR4 expressed by hematopoietic cells and the IL-1 receptor on radioresistant cells (25). Studies from our laboratory on carcinogenic polyaromatic hydrocarbon 7,12-dimethylbenz(a)anthracene (DMBA) have demonstrated that cell mediated immunity to DMBA was dependent on TLR4 and had a protective effect against mammary tumor development. This effect was primarily mediated by IL-12 secreted by CD11c+ cells in TLR4 proficient mice, which lead to an IFN-γ mediated response resulting in fewer tumors (26).

## Toll-Like Receptors and Immunotherapy of Breast Cancer

Vaccination strategies using DC/breast carcinoma fusions were effective in generating anti-tumor immune responses patients with metastatic breast cancer, but tumor regression was observed in a minor group of these patients. This was due to the expansion of both activated and regulatory T-cell populations by DC/breast carcinoma fusions, primarily leading to suppression of T-cell responses. TLR9 agonist CpG oligodeoxynucleotides along with IL-12 and IL-18 was able to reduce the level of fusion-mediated regulatory T-cell expansion. The regulatory T-cell response was inhibited by using TLR agonists that enhanced effector T-cell responses, thus increasing the efficacy of vaccine (27). Effective immunotherapy using combination of HER-2/neu genetic vaccine and novel agonist of TLR9 has been reported for breast cancer. This therapy has been reported to be associated with antibody isotype switch and antibody-dependent cellular cytotoxicity activity of the of DNA-EP/Ad-based cancer vaccines (28). Vaccination with Ad-BD2-E1A (E1B-deleted oncolytic adenovirus expressing beta-defensin-2) vaccine inhibited primary breast tumor growth and blocked metastasis in a TLR4 dependent manner, thus suggesting the critical role of TLR4 in the induction of anti-tumor immunity by Ad-BD2-E1A (29).

## Toll-Like Receptors and Chemotherapy of Breast Cancer

The efficacy of chemotherapy is defined by their ability to perturb the division of tumor cells. A successful outcome of chemotherapy or radiotherapy also involves inclusion of an adjuvant that would enhance the efficacy of chemo- or radiotherapy. One such adjuvant, high mobility group box 1 protein (HMGB1) has been successfully used in therapy of breast cancer. Dying tumor cells release HMGB1 that has been shown to activate TLR4 on DCs. It was shown that TLR4 expressed on DCs was required for the cross presentation of tumor antigens and the promotion of tumor specific cytotoxic T-cell responses. Breast cancer patients harboring the loss-of-function Asp299Gly polymorphism of TLR4 relapsed earlier after receiving anthracycline-based chemotherapy. These data suggests that HMGB1- and TLR4-dependent immune responses elicited by conventional cancer treatment may increase the probability of achieving a lasting therapeutic success (30). When small nucleotide polymorphisms (SNPs) in TLR2, TLR3, TLR4, and TLR9 were assessed for their association with breast cancer, no association was found. However, population genetics data has revealed that a hypomorphic variant of TLR4 (p.Asp299Gly) allele was found with no specific allelic frequency (8.4%) in the Croatian population compared to other Caucasians (6.5–10%) (31). The development of drugs targeting TLRs is an emerging area, and there are about 20 drugs that are in pre-clinical and clinical trials (32).

## Toll-Like Receptors and Breast Cancer Cells

In addition to their expression on immune cells, TLRs are also expressed on tumor cells (15). Activation of TLR expressed on tumor cells may enhance tumor growth by increasing pro-survival signals, anti-apoptotic signals, tumor promoting cytokines, angiogenesis, and invasiveness (33, 34). Among the TLRs (TLR1-10), expressed on human breast cancer cell line MDA-MB-231, expression of TLR4 was found to be the highest, and knockdown of TLR4 gene resulted in significant cell death and inhibition of IL-6 and IL-8 cytokines, compared with vector control (33). In another study, TLR9 was shown to increase invasion of MDA-MB-231 cells, by increasing the activity of matrix metalloproteinase 13 (MMP13) (35). TLR3, TLR4, and TLR9 have been shown to be highly expressed in human breast tumors. There was also an increase in the expression of TLR4 by mononuclear inflammatory cells and TLR9 by fibroblast-like cells in mammary tumors. There was more metastasis in TLR3 expressing tumor cells, TLR4 expressing inflammatory cells but not in TLR9 expressing fibroblasts like cells (36). TLR9 isoforms A and B has been detected in clinical breast cancer, and ERα and sex steroid hormones have been shown to contribute to its invasiveness. TLR9 expression was also found to be affected by the hormonal cancer therapy using bicalutamide (37). In a randomized clinical trial using poly (A:U) dsRNA, TLR3 agonist, chemotherapy was enhanced in patients with TLR3-positive cancers. Chemotherapy using poly A:U was successful only when it was combined with an immunochemotherapeutic regimen of vaccination against tumor antigens (38). In a recent study, it was found that activation of TLR5 on breast cancer cells by its agonist flagellin, led to inhibition of cell proliferation and anchorage dependent cell growth. This was further confirmed in mouse xenograft models using human breast cancer cells. This inhibitory activity was further confirmed *in vivo* using mouse xenografts of human breast cancer cells (39). Inflammatory signals generated by TLR signaling have also been reported to increase expression of chemokines, thus causing an influx of Th17 cells by tumor cells and tumor derived fibroblasts (40).

## Conclusions and Perspectives

**Figure 1:**
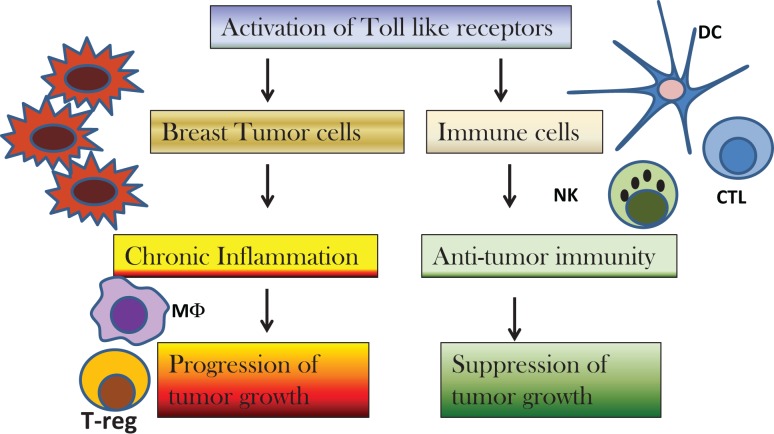
**Diagram to depict the effect of activation of toll-like receptors (TLRs) on breast cancer cells versus immune cells**. Activation of TLRs on breast cancer cells results in chronic inflammation and recruitment of macrophages (MΦ) and regulatory T-cells (T-reg) in the tumor microenvironment that cause suppression of immune responses and progression of tumor growth. Activation of TLRs by TLR agonists cause infiltration of dendritic cells (DC), natural killer (NK) cells, and cytotoxic T-cells (CTL) that result in suppression of tumor growth.

Discovery of the role of TLRs in cancer biology have paved the way for development of new therapies targeting TLRs. There is a lot of interest to study the relation between inflammation and cancer as it has been termed as the seventh hallmark of cancer. TLRs play an important role in inflammation mediated cancers as well as cancer related inflammation. Activation of TLRs for therapy may be an exciting proposition, but one has to be careful as over activation of TLRs can also lead to development of tumors (Figure 1). Thus, regulatory mechanisms should also be taken into account before using TLRs for cancer therapy. Furthermore, molecular and genetic analysis of breast cancer sub-types should be considered before deciding the course of therapy with TLRs. There are some reports on the role of genetic polymorphisms in TLRs in the outcome of breast cancer therapy. More studies need to be conducted to determine whether the loss or gain of function polymorphisms in TLRs is an indicator of disease outcome or therapy.

## Conflict of Interest Statement

The author declares that the research was conducted in the absence of any commercial or financial relationships that could be construed as a potential conflict of interest.
